# Celloidin in Surgery

**Published:** 1905-02

**Authors:** 


### ABSTRACT.

### Celloidin in Surgery.

Dr. Frederick Holme Wiggin, New York, (*International Journal of Surgery*, May, 1904,) details the technic of an appendicitis operation. After suturing the wound and disinfecting the skin, he applies the following solution:

| | | |
|---|---|---|
| R | Alcohol, 96 per cent. | |
| | Ether | āā 49½ ozs. |
| | Celloidin | 1 oz. |
| | Add ol. ricini | ½ oz. |
| M. et ft. sol. | | |

This thoroughly seals the wound, but permits of its complete inspection, as the celloidin is transparent. The dressing also acts as a splint and holds the edges of the wound in proper position. If properly applied, it prevents infection of the wound and tissues from displacement of the bandage or careless handling. This is a great advantage, especially when the surgeon is obliged after the operation to leave the patient during convalescence to the care of others.

The preparation was first called to his attention by Mr. E. Stanmore Bishop, of Manchester, England It must not be applied too liberally, as it has strong contractile powers and if too large a surface is covered it is likely to contract sufficiently to cause the edges of the wound to turn inward. The solution should be made fresh for each operation, as ether and absolute alcohol evaporate rapildy. Care must be exercised to see that the skin is well dried and the wound is not oozing, or the solution will not adhere properly. A piece of gauze is placed over the dressing and held in place with adhesive strips lightly applied, so as to make no pressure. After four or five days if there are no signs of infection, this can be removed. When properly applied, the celloidin will adhere for eleven or twelve days; but if there is an infection it will be found loose and can be removed like an old scab.

Dr. Wiggin has used this dressing for five or six years and has found it very satisfactory and a great aid in his surgical work.

